# The genetic and environmental structure of the character sub-scales of the temperament and character inventory in adolescence

**DOI:** 10.1186/s12991-016-0094-2

**Published:** 2016-03-12

**Authors:** Nigel Lester, Danilo Garcia, Sebastian Lundström, Sven Brändström, Maria Råstam, Nóra Kerekes, Thomas Nilsson, C. Robert Cloninger, Henrik Anckarsäter

**Affiliations:** Department of Psychiatry, Center for Well-Being, Washington University School of Medicine, St. Louis, MO USA; Blekinge Center of Competence, Blekinge County Council, Karlskrona, Sweden; Department of Psychology, University of Gothenburg, Gothenburg, Sweden; Department of Psychology, Lund University, Lund, Sweden; Network for Empowerment and Well-Being, Lyckeby, Sweden; Institute for Neuroscience and Physiology, Centre for Ethics, Law and Mental Health (CELAM), University of Gothenburg, Gothenburg, Sweden; R&E unit, Swedish Prison and Probation Service, Norrköping, Sweden; Gillberg Neuropsychiatry Centre, Institution of Neuroscience and Physiology, University of Gothenburg, Gothenburg, Sweden; Department of Clinical Sciences, Lund University, Lund, Sweden; Institution for Health Sciences, University West, Trollhättan, Sweden

**Keywords:** Adolescence, CATSS, Cloninger’s psychobiological model, Cooperativeness, Genetics, Personality, Self-directedness, Self-transcendence, Sub-scales, Temperament, Character inventory

## Abstract

**Background:**

The character higher order scales (self-directedness, cooperativeness, and self-transcendence) in the temperament and character inventory are important general measures of health and well-being [Mens Sana Monograph 11:16–24 (2013)]. Recent research has found suggestive evidence of common environmental influence on the development of these character traits during adolescence. The present article expands earlier research by focusing on the internal consistency and the etiology of traits measured by the lower order sub-scales of the character traits in adolescence.

**Methods:**

The twin modeling analysis of 423 monozygotic pairs and 408 same sex dizygotic pairs estimated additive genetics (A), common environmental (C), and non-shared environmental (E) influences on twin resemblance. All twins were part of the on-going longitudinal Child and Adolescent Twin Study in Sweden (CATSS).

**Results:**

The twin modeling analysis suggested a common environmental contribution for two out of five self-directedness sub-scales (0.14 and 0.23), for three out of five cooperativeness sub-scales (0.07–0.17), and for all three self-transcendence sub-scales (0.10–0.12).

**Conclusion:**

The genetic structure at the level of the character lower order sub-scales in adolescents shows that the proportion of the shared environmental component varies in the trait of self-directedness and in the trait of cooperativeness, while it is relatively stable across the components of self-transcendence. The presence of this unique shared environmental effect in adolescence has implications for understanding the relative importance of interventions and treatment strategies aimed at promoting overall maturation of character, mental health, and well-being during this period of the life span.

**Electronic supplementary material:**

The online version of this article (doi:10.1186/s12991-016-0094-2) contains supplementary material, which is available to authorized users.

## Background

Cloninger’s theory of personality proposes that human beings are comprised of an integrated hierarchy of biological, psychological, and social systems that allow them to adapt more or less flexibly and maturely to changes in their external and internal milieu [1]. This model consists of a temperament domain (i.e., individual differences in behavioral learning mechanisms influencing basic emotional drives) and a character domain (i.e., self-concepts about goals and values that express what people make of themselves intentionally). For the measurement of these personality domains, Cloninger has developed the temperament and character inventory [1] composed of four dimensions of temperament (novelty seeking, harm avoidance, reward dependence, and persistence) and three dimensions of character (self-directedness, cooperativeness, and self-transcendence). These temperament and character dimensions serve as tools for disentangling personality profiles of healthy individuals, as well as of individuals with neuropsychiatric disorders [2–6]. Moreover, self-directedness, cooperativeness, and self-transcendence assessed by the Temperament and Character Inventory are important general measures of health and well-being [7–10]. Self-transcendence, however, is positively related to both positive and negative emotions during the adolescent years and during adulthood in cultures that discourage open emotional expression [4, 11].

**Fig. 1:**
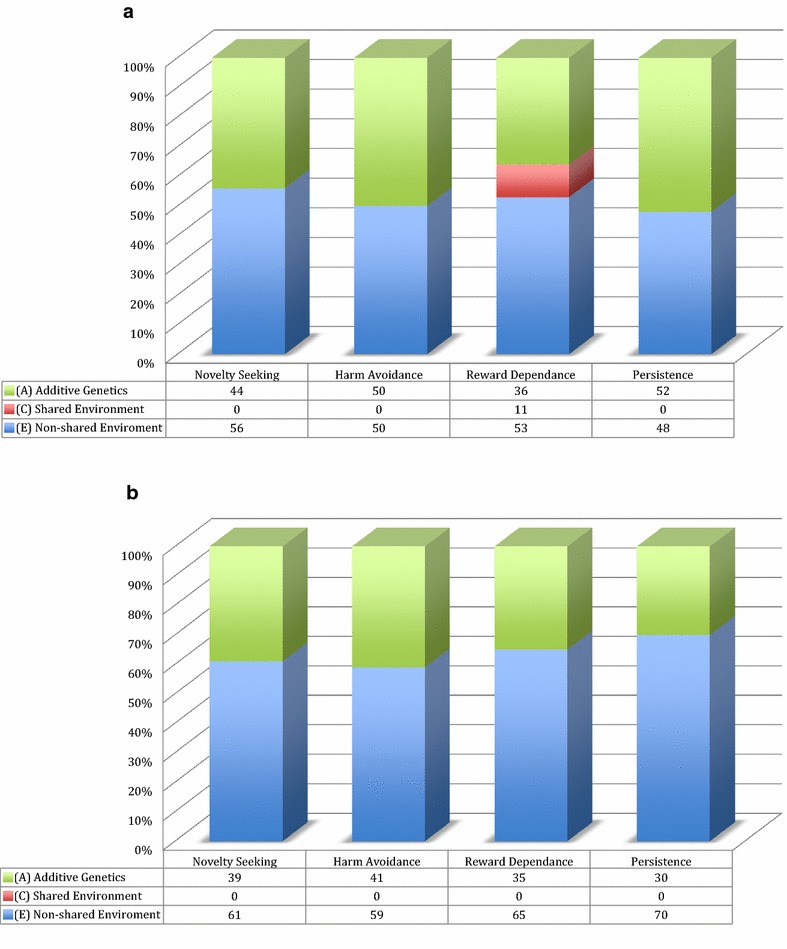
The effect sizes of additive genetics (*A*) and non-shared environmental effect (*E*) across the temperament scales in (**a**) adolescents [14] compared to (**b**) adults [12]

**Fig. 2:**
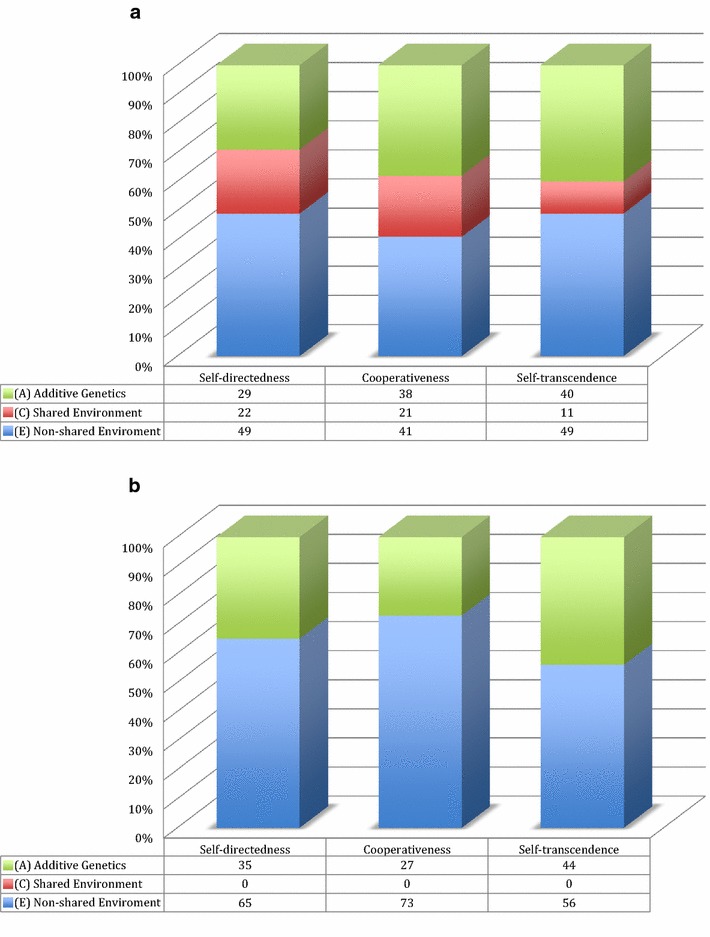
The effect sizes of additive genetics (*A*), shared environment (*C*), and non-shared environmental effect (*E*) across the character higher order scales in (**a**) adolescents [14] compared to (**b**) adults [12]

Previous findings have shown that heritability influences on character are about the same across studies using different age groups. Nonetheless, there are some differences worth noting. For example, while the character scales do not show common environmental influences in research among older adults (e.g., [12]), a small common environmental influence for self-directedness and cooperativeness has been found among young adults (20–30 years of age; e.g., [13]). In addition, recent research using one of the largest population-based twin studies among adolescents, found suggestive evidence of common environmental influence for all of the character scales [14]. What is more, Gillespie and colleagues [12] showed in adults, and Garcia and colleagues [14] in adolescents, that the genetic structure of the temperament higher order scales shows no evidence of a shared or common environmental effect (C) across the scales. The exception being that in adolescents, in contrast to adults, there was a small shared environmental effect in the temperament dimension of reward dependence (i.e., the individuals' tendency to respond markedly to signals of social approval, social support, and sentimentality). The effect size is similar to that which is observed in adolescents’ character dimensions. Overall the effect size of additive genetics (A) to non-shared environmental effect (E) is slightly larger across the temperament dimensions in adolescents compared to adults (see Fig. 1a, b). In contrast, the genetic structure of the character scales in the adolescent sample shows a modest but noteworthy proportion of shared environmental influence that is not present in the adult sample studied by Gillespie and colleagues (Fig. 2a, b). In other words, there is greater consistency, between the adolescent and the adult sample, in the proportion of additive genetic effect to non-shared environmental effect with respect to temperament but not with respect to character. These results suggest a “shift” in the type of environmental influence (i.e., shared to non-shared) from adolescence to adulthood with regard to character. In this context, it is important to point out that interventions to enhance self-directedness and cooperativeness can alleviate dysfunction and suffering related to different psychiatric disorders [3]. Character traits improve with cognitive-behavioral treatments and baseline levels of character are strong predictors of clinical outcomes [15–18]. If the “shift” in environmental influence exists, then interventions targeting character development may be more successful if conducted during adolescence or young adulthood.

The Temperament and Character Inventory’s character scales, as well as those scales measuring temperament, are higher order scales composed of lower order sub-scales. The higher order scales have the advantage of allowing the prediction of many outcomes (e.g., personality disorders) because they represent wide-ranging descriptions of personality (see [19]). Nevertheless, one disadvantage when personality is only investigated in terms of broad scales is that the aggregation of the lower order sub-scales in one higher order scale results in a loss of information—information that might be useful for psychological description, prediction, and explanation (see [19]). The present article expands earlier research (e.g., [14]) by focusing on the etiology of the lower order sub-scales of the character dimension of personality in adolescence. Thus, it targets information that may be useful in the study of adolescents’ mental health and well-being. For a brief description of low and high scorers in each of the lower order sub-scales for the character traits of the Temperament and Character Inventory, please see the Tables 1, 2, and 3.

**Table 1:** The five lower order sub-scales that compose the self-directedness (SD) scale of the Temperament and Character Inventory

High Scorers		Low Scorers
Tend to feel free to choose what they will do. They recognize that their attitudes, behaviors, and problems generally reflect their own choices. Consequently, they tend to accept responsibility for their attitudes and behavior. They are reliable and trustworthy	(SD1) responsibility vs. blaming	Tend to blame other people and external circumstances for what is happening to them. They feel that their attitudes, behavior, and choices are determined by influences outside their control or against their will. Consequently, they tend not to accept responsibility for their actions
They have a clear sense of meaning and direction in their lives. They have developed the ability to delay gratification to achieve their goals	(SD2) purposefulness vs. lack of goal direction	Tend to struggle to find direction, purpose, and meaning in their lives. They are uncertain about long-term goals, and thus feel driven to react to current circumstances and immediate needs. They may feel that their life is empty and has little or no meaning beyond the reactive impulses of the moment
Usually described as resourceful and effective. They impress other people as productive, proactive, competent, and innovative individuals who rarely lack ideas on how to solve problems or initiative in identifying opportunities to solve problems. Indeed, they tend to look at a difficult situation as a challenge or an opportunity	(SD3) resourcefulness vs. inertia	Impress others as helpless, hopeless, and ineffective. These individuals have not developed skills and confidence in solving problems and thus feel unable and incompetent when faced with obstacles. Typically, they tend to wait for others to take the lead in getting things done
Self-confident individuals who recognize and accept both their strengths and limitations. In other words, these individuals try to do the best that they can without pretending to be something they are not. Rather, they seem to accept and feel very comfortable with their actual mental and physical features, although they may try to improve these limitations by constructive training and effort	(SD4) self-acceptance vs. self-striving	Tend to manifest low self-esteem. They neither accept nor enjoy their actual mental and physical features. Rather, they often pretend to be different than they really are. That is, they tend to fantasize about unlimited wealth, importance, beauty, and perpetual youth. When confronted with evidence to the contrary, they may become severely disturbed
These individuals have developed a spectrum of goal-congruent, good habits so that they automatically act in accord with their long-term values and goals. This is achieved gradually as a consequence of self-discipline, but eventually becomes automatic. These habits usually develop through repeated practice and are typically stronger than most momentary impulses or persuasion. In other words, these individuals rarely confuse their priorities and thus feel safe and self trusting in many tempting situations	(SD5) self-actualizing vs. bad habits	These individual manifest habits that are inconsistent with and make it hard for them to accomplish worthwhile goals. Others sometimes perceive these peoples as self-defeating and weak-willed. In other words, their will power appears to be too weak to overcome many strong temptations, even if they know that they will suffer as a consequence

**Table 2:** The five lower order sub-scales that compose the cooperativeness (CO) scale of the Temperament and Character Inventory

High Scorers		Low Scorers
They are described as tolerant and friendly. These individuals tend to accept other people as they are, even people with very different behaviors, ethics, opinions, values, or appearances	(CO1) social acceptance vs. social intolerance	They are typically impatient with and critical of other people, especially people who have different goals and values
They typically try to imagine themselves “in other people’s shoes”. In other words, these individuals are highly attenuated to and considerate of other people’s feelings. They tend to treat others with dignity and respect, and often put aside their own judgement initially so they can better understand what other people are experiencing. Empathy also involves a conscious understanding of, and respect for, the goals and values of other people	(CO2) empathy vs. social disinterest	These individuals do not seem to be very concerned about other’s feeling. Rather they seem to be unable to share in another’s emotions, suffering, or hardship, or at least are unwilling to respect (i.e., assign value to) the goals and values of other people
Tend to be helpful, supportive, encouraging, or reassuring. These individuals enjoy being in service of others. Often they share their skills and knowledge so that everyone comes out ahead. They like to work as part of a team, usually preferring this to working alone	(CO3) helpfulness vs. unhelpfulness	They are described as self-centerd, egoistic, or selfish. They tend to be inconsiderate of other people and typically look out only for themselves, even when working in a team of highly cooperative collaborators. They prefer to work alone or to be in charge of what is done
These individuals are described as compassionate, forgiving, charitable, and benevolent. They do not enjoy revenge and usually do not try to get even if they were treated badly. Rather, these individuals actively try to get over insults or unfair treatment in order to be constructive in a relationship	(CO4) compassion vs. revengefulness	Tend to enjoy getting revenge on people who hurt them. Their revengeful triumph can be either overt or disguised. The former is observed as active-aggressive behavior, such as hurting other physically, emotionally, and financially. The latter is observed as passive-aggressive behaviors, such as holding grudges, deliberate forgetfulness, stubbornness, and procrastination
They are described as honest, genuinely scrupulous, and sincere persons who treat others in a consistently fair manner. In other words, these persons have incorporated stable ethical principles and scruples in both their professional and their social and interpersonal relationships. Such ethical standards are a component of social cooperativeness, rather than related to self-Transcendence or spirituality	(CO5) integrated conscience vs. self-serving advantage	These individuals are described as opportunistic, i.e., they would do whatever they can to get away with to reach their goals without getting in immediate trouble. These individuals tend to treat other people unfairly, in a biased, self-serving manner that usually reflects their own profit. They are thus frequently described as manipulative or deceitful. In other words, they have not incorporated stable ethical principles and scruples into their social and interpersonal relationships

**Table 3:** The three lower order sub-scales that compose the self-transcendence (ST) scale of the Temperament and Character Inventory

High Scorers		Low Scorers
Tend to transcend their self-boundaries when deeply involved in a relationship or when concentrating in what they are doing, forget where they are for a while and lose awareness of the passage of time. Thus, appearing “in another world” or “absent minded”. Individuals who experience such self-forgetfulness are often described as creative and original	(ST1) creative self-forgetfulness vs. self-conscious experience	Tend to remain aware of their individuality in a relationship or when concentrating on their work. These individuals are rarely deeply moved by art or beauty. Thus, others usually perceive them as conventional, prosaic, unimaginative, or self-conscious
Tend to experience an extraordinarily strong connection to nature and the universe as a whole, including the physical environment as well as people. They often report feeling that everything seems to be a part of a living organism and are often willing to make personal sacrifices in order to make the world a better place by trying to prevent war, poverty, or injustice. They might be regarded as fuzzy-thinking idealists	(ST2) transpersonal identification vs. personal identification	Rarely experience strong connections to nature or people. They tend to be individualists who feel that they are neither directly nor indirectly responsible for what is going on with other people or the rest of the world. Such individuals view nature as an external object to be manipulated instrumentally, rather than something of which they are an integral part
Often believe in miracles, extrasensory experiences, and other spiritual phenomena such as telepathy or a “sixth sense”. They show magical thinking and are both vitalized and comforted by spiritual experiences. They might deal with suffering and even death through faith that they have, which may involve communion with their God	(ST3) spiritual acceptance vs. rational materialism	Tend to accept only materialism and objective empiricism. They are often unwilling to accept things that cannot be scientifically explained. This, in turn, is a disadvantage when they face situations over which there is no control or possibility for evaluating by rational objective means (e.g., inevitable death, suffering, or unjust punishments)

### The present study

The present study expands earlier research by focusing on the internal consistency and the etiology of traits measured by the lower order sub-scales of the character traits in adolescence. The study was conducted using self-reported character measures from The Child and Adolescent Twin Study in Sweden (CATSS), which is an on-going large population-based longitudinal twin study targeting all twins born in Sweden since July 1, 1992. By January 2013, the CATSS comprised around 23,000 twins and it had a response rate of roughly 76 % (for a detailed description of the CATSS see [20]). We used data from a sample of 15-year-old twins (detailed in [14]) in order to capture a critical period of life where personality undergoes huge developmental processes related to adolescents’ ill- and well-being. We target the etiology of the character sub-scales to catch information that may be useful for psychological description, prediction, and explanation of mental health and well-being.

## Methods

### Ethical statement

The present analyses included twins who provided data at the CATSS-9/12, CATSS-15, and DOGSS studies. All data collections have separate ethical approvals from the Karolinska Institute ethical review board (DNR: 02-289, 2010/597-31/1, 2010/1356/31/1, 03-672, and 2009/739-31/5). The participants, both parent and children/adolescents are protected by informed consent process. They were informed of what is being collected and were repeatedly given the option to withdraw their consent and discontinue their participation.

### Sample and procedure

In the present study we used data from the CATSS, earlier described in Garcia et al. [14], whose parents were interviewed by telephone using the Autism—Tics, ADHD ,and other Comorbidities inventory [21] when the twins were 9 or 12 years of age. At the age of 15, the twins completed a battery of questionnaires that were sent by mail (overall response rate 48 %), including the short version (125 items) of the Temperament and Character Inventory. Moreover, twins who screened positive for any neuropsychiatric disorder and controls were part of a detailed clinical interview that included the longer Temperament and Character Inventory version (238 items).^1^ Previously, Garcia and colleagues [14] developed a valid and reliable item-extraction procedure to generate the short version from the larger version of temperament and character inventory. This allowed us to conduct the correlation, reliability, and the twin modeling analysis using the whole twin sample based on the short version of the Temperament and Character Inventory. Only twins who had a maximum of 5 % missed items and have answered the control questions correctly were included in the final analyses (a common procedure regarding the Temperament and Character Inventory, [22]).

For the correlation and reliability analysis, addressing the internal consistency of the lower order sub-scales (Additional file 1: Tables S1–S3), we used a total of 2714 twins (878 monozygotic, 885 same sex dizygotic, 638 different sex dizygotic, and 313 of unknown zygosity). The twin modeling analysis addressing the etiology of traits measured by the lower order sub-scales of the character traits required only twins with known zygosity. In essence the model compares traits in monozygotic twins, who are genetically identical, with traits in dizygotic twins, who on average share 50 % of their segregating alleles. The difference in genetic relatedness can then be used to disentangle the genetic and environmental contribution to a trait, in this case the lower order character traits. Hence, for this specific analysis we were only able to use 423 monozygotic pairs and 408 same sex dizygotic pairs.

### Measures

#### Zygosity

Zygosity was determined on the basis of 48 single nucleotide polymorphisms [20]. For twins without available DNA, zygosity was determined using a validated algorithm based on five questions on twin similarity derived from 571 pairs of twins with known zygosity. Only twins with more than 95 % probability of being correctly classified, compared to DNA testing, were assigned zygosity by this method. In other words, the twins with less than 95 % probability of being correctly classified were assigned as unknown zygosity [23].

#### Temperament and Character Inventory

The temperament and character inventory measures the seven scales, and its sub-scales, of the psychobiological model of personality (binary answer: *true* = 1, *false* = 0). The five sub-scales of the self-directedness scales are: responsibility vs. blaming (SD1, e.g., “I often feel that I am the victim of circumstances”, reverse coded), purposefulness vs. lack of goal direction (SD2, e.g., “My behavior is strongly guided by certain goals that I have set for my life”), resourcefulness vs. inertia (SD3, e.g., “I usually look at a difficult situation as a challenge or opportunity”), self-acceptance vs. self-striving (SD4, e.g., “I often wish I was stronger than everyone else”, reverse coded), and self-actualization (former congruent second nature) vs. bad habits (SD5, e.g., “Many of my habits make it hard for me to accomplish worthwhile goals”, reverse coded).

The five sub-scales of the cooperativeness scales are: social acceptance vs. social intolerance (CO1, e.g., “I can usually accept other people as they are, even when they are very different from me”), empathy vs. social disinterest (CO2, e.g., “I often consider another person’s feelings as much as my own”), helpfulness vs. unhelpfulness (CO3, e.g., “I like to share what I have learned with other people”), compassion vs. revengefulness (CO4, e.g., “I hate to see anyone suffer”), and integrated conscience vs. self-serving advantage (CO5, e.g., “I cannot have any peace of mind if I treat other people unfairly, even if they are unfair to me”).

The three sub-scales of the self-transcendence scales are: creative self-forgetfulness vs. self-conscious experience (ST1, e.g., “I often become so fascinated with what I’m doing that I get lost in the moment—like I’m detached from time and place”), transpersonal identification vs. personal identification (ST2, e.g., “I sometimes feel so connected to nature that everything seems to be part of one living organism”), and spiritual acceptance vs. rational materialism (ST3, e.g., “I seem to have a “sixth sense” that sometimes allows me to know what is going to happen”).

### Statistical treatment

All data were considered to be normally distributed after graphical exploration (histograms), thus all statistical tests were conducted using parametric methods in SPSS version 19. *Cronbach ‘s alphas* and *Pearson’s correlations coefficients* for the character lower order sub-scales are reported in Additional file 1: Table S1–S3.

The etiology of the character lower order sub-scales was investigated using twin methodology. The genetic and environmental contributions are portioned into three variance components: additive genetic factors (A), common environmental factors that make the twins similar (C), and unique environmental factors that make the twins dissimilar (E). In the first step, intraclass correlation (ICC) coefficients, for the character sub-scales, were calculated separately for monozygotic twins and same sex dizygotic twins. As a second step, we performed univariate genetic analyses, using a model-fitting approach with structural equation-modeling techniques conducted in Mx [24].

## Results

The correlation and reliability analysis addressing the internal consistency of the lower order sub-scales is presented in Additional file 1: Table S1–S3. The twin modeling analysis addressing the etiology of traits suggested a common environmental contribution for the following self-directedness sub-scales: purposefulness vs. lack of goal direction (0.14) and self-actualizing (former congruent second nature) vs. bad habits (0.23); for three of the cooperativeness sub-scales: empathy vs. social disinterest (0.10), helpfulness vs. unhelpfulness (0.07), and compassion vs. revengefulness (0.17); and for all three self-transcendence sub-scales: creative self-forgetfulness vs. self-conscious experience, transpersonal identification vs. personal identification, and spiritual acceptance vs. rational materialism (between 0.10 and .12). All sub-scales in the self-directedness scale were under a large unique environmental influence that ranged from 0.49 to 0.70 (Table 4). This pattern could be discerned in all sub-scales in the cooperativeness (Table 5) and self-transcendence (Table 6) scales as well. There was a general trend suggesting that the genetic component had a larger influence than the common environmental component, in all sub-scales of the three character dimensions. The confidence intervals were, however, overlapping in all cases.

**Table 4:** Intraclass correlations (ICC) according to zygosity and estimates of genetic and environmental effects for the five lower order sub-scales that compose the self-directedness scale of the Temperament and Character Inventory [95 % confidence interval]

	MZ	DZ	A	C	E
	(*n* = 423 pairs)	(*n* = 408 pairs)	Additive genetics	Common environment	Unique environment
Self-directedness	0.52	0.36	.29	.22	.49
	[.44, .58]	[.27, .44]	[.09, .50]	[.04, .38]	[.43, .56]
(SD1) responsibility vs. blaming	0.45	0.21	0.42	0.01	0.57
	[.37, .52]	[.12, .31]	[.19, .50]	[.00, .21]	[.50, .64]
(SD2) purposefulness vs. lack of goal direction	0.30	0.22	0.16	0.14	0.70
	[.21, .38]	[.12, .31]	[.00, .38]	[.00, .31]	[.62, .79]
(SD3) resourcefulness vs. inertia	0.40	0.23	0.32	0.07	0.61
	[.32, .48]	[.13, .32]	[.08, .46]	[.00, .27]	[.54, .69]
(SD4) self-acceptance vs. self-striving	0.48	0.21	0.47	0.00	0.53
	[.41, .55]	[.11, .30]	[.30, .53]	[.00, .14]	[.47, .60]
(SD5) self-actualizing vs. bad habits	0.33	0.29	0.11	0.23	0.66
	[.23, .41]	[.20, .38]	[.00, .35]	[.03, .36]	[.58, .75]

*MZ* monozygotic, *DZ* dizygotic

**Table 5:** Intraclass correlations (ICC) according to zygosity and estimates of genetic and environmental effects for the five lower order sub-scales that compose the cooperativeness scale of the Temperament and Character Inventory [95 % confidence interval]

	MZ	DZ	A	C	E
	(*n* = 423 pairs)	(*n* = 408 pairs)	Additive genetics	Common environment	Unique environment
Cooperativeness	0.59	0.40	.38	.21	.41
	[.52, .65]	[.32, .48]	[.19, .57]	[.04, .37]	[.36, .47]
(CO1) social acceptance vs. social Intolerance	0.40	0.22	0.42	0.00	0.58
	[.32, .48]	[.12, .31]	[.20, .49]	[.00, .18]	[.51, .66]
(CO2) empathy vs. social disinterest	0.41	0.25	0.30	0.10	0.60
	[.31, .47]	[.16, .34]	[.06, .47]	[.00, .29]	[.53, .68]
(CO3) helpfulness vs. unhelpfulness	0.35	0.20	0.27	0.07	0.66
	[.27, .43]	[.11, .29]	[.03, .42]	[.00, .27]	[.58, .74]
(CO4) compassion vs. revengefulness	0.51	0.36	0.36	0.17	0.47
	[.44, .58]	[.27, .44]	[.16, .56]	[.00, .33]	[.41, .55]
(CO5) integrated conscience vs. self-serving advantage	0.34	0.16	0.33	0.00	0.67
	[.25, .42]	[.06, .25]	[.11, .41]	[.00, .18]	[.59, .75]

*MZ* monozygotic, *DZ* dizygotic

**Table 6:** Intraclass correlations (ICC) according to zygosity and estimates of genetic and environmental effects for the three lower order sub-scales that compose the self-transcendence scale of the Temperament and Character Inventory [95 % confidence interval]

	MZ	DZ	A	C	E
	(*n* = 423 pairs)	(*n* = 408 pairs)	Additive genetics	Common environment	Unique environment
Self-transcendence	0.51	0.31	.40	.11	.49
	[.43, .58]	[.22, .39]	[.19, .56]	[.00, .28]	[.43, .56]
(ST1) creative self-forgetfulness vs. self-conscious experience	0.42	0.27	0.32	0.11	0.57
	[.34, .50]	[.17, .36]	[.09, .49]	[.00, .29]	[.50, .65]
(ST2) transpersonal Identification vs. personal identification	0.41	0.26	0.31	0.10	0.59
	[.33, .48]	[.17, .35]	[.08, .49]	[.00, .29]	[.51, .67]
(ST3) spiritual acceptance vs. rational materialism	0.46	0.27	0.33	0.12	0.55
	[.38, .53]	[.18, .36]	[.11, .51]	[.00, .30]	[.49, .63]

*MZ* monozygotic, *DZ* dizygotic

## Discussion

In the introduction section we have detailed the differences between adolescents and adults in the genetic structure of temperament and character dimensions of personality. These differences suggest a “shift” in the type of environmental influence (i.e., shared to non-shared) from adolescence to adulthood with regard to character. Our study looks in greater depth at these variations, in particular the evidence for a shared environmental effect on the lower order sub-scales of the character traits during adolescence.

In their study among older adults, Gillespie and colleagues [12] expected shared environmental effects to account for a significant proportion in character variance because character traits were earlier hypothesized by Cloninger [25] to be partly due to socio-cultural learning. Nevertheless, these researchers found that additive genetic effects alone provided the most parsimonious explanation for the source of familial aggregation in each character higher order scale. Based on their univariate analysis, genetic effects explained 27–44 % of the variance in the three character higher order scales. Despite limitations of power in their study, the rejection of an ACE model in favor of AE was consistent with other studies in adult populations [26, 27].

In contrast to this evidence from adults, we provide evidence to support the role of shared environmental effects (C) on character variability in adolescence. Our findings support an ACE model and are therefore more consistent with earlier theoretical expectations [25] and recent empirical findings about the important influence of parental rearing and cultural norms on character development [28, 29]. The importance of both the underlying biological and social determinants during this critical phase of personality development are therefore likely to be critical in character maturation. It may be that this common environmental effect supported by our study in adolescents operates primarily in early development or that the methodology is concealing the effect in adults.

**Fig. 3:**
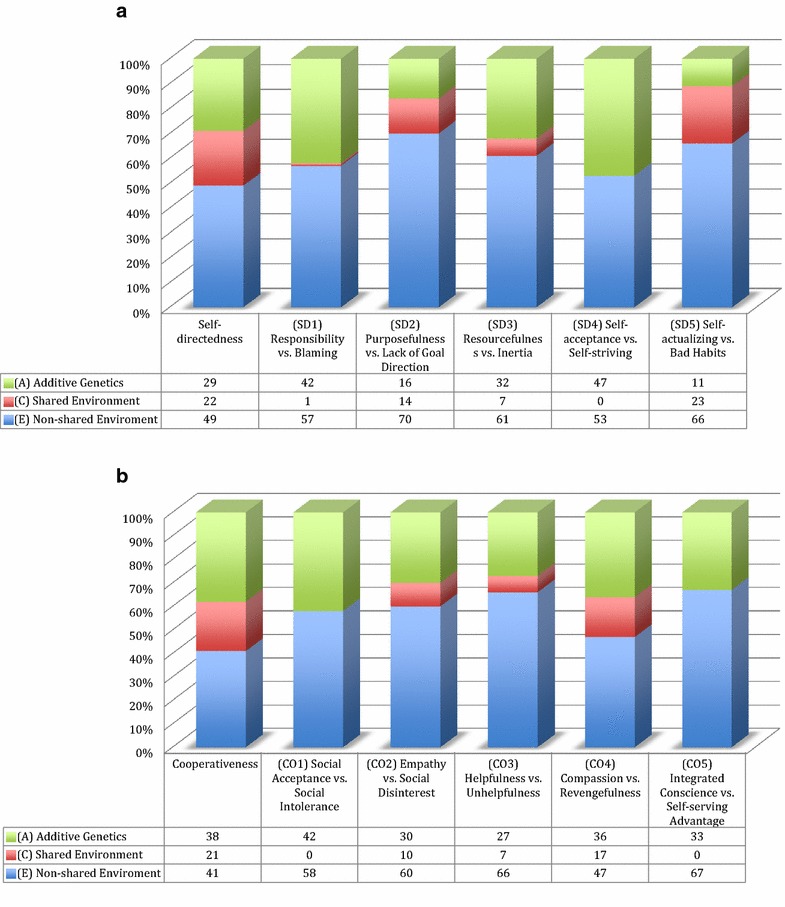
The effect sizes in the present study of additive genetics (*A*), shared environment (*C*), and non-shared environmental effect (*E*) across the character lower order sub-scales of **a** self-directedness and **b** cooperativeness

**Fig. 4:**
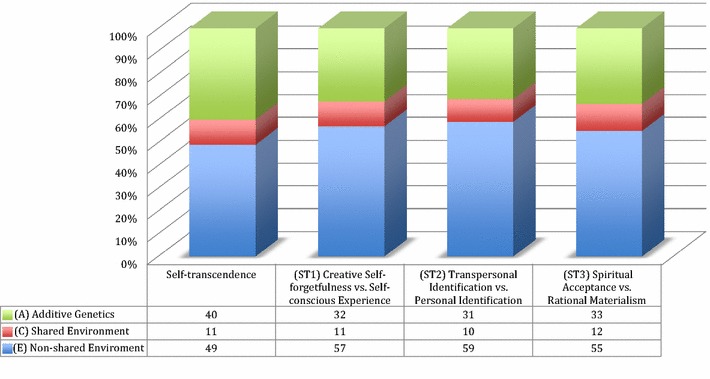
The effect sizes in the present study of additive genetics (*A*), shared environment (*C*), and non-shared environmental effect (*E*) across the character lower order sub-scale of self-transcendence

**Fig. 5:**
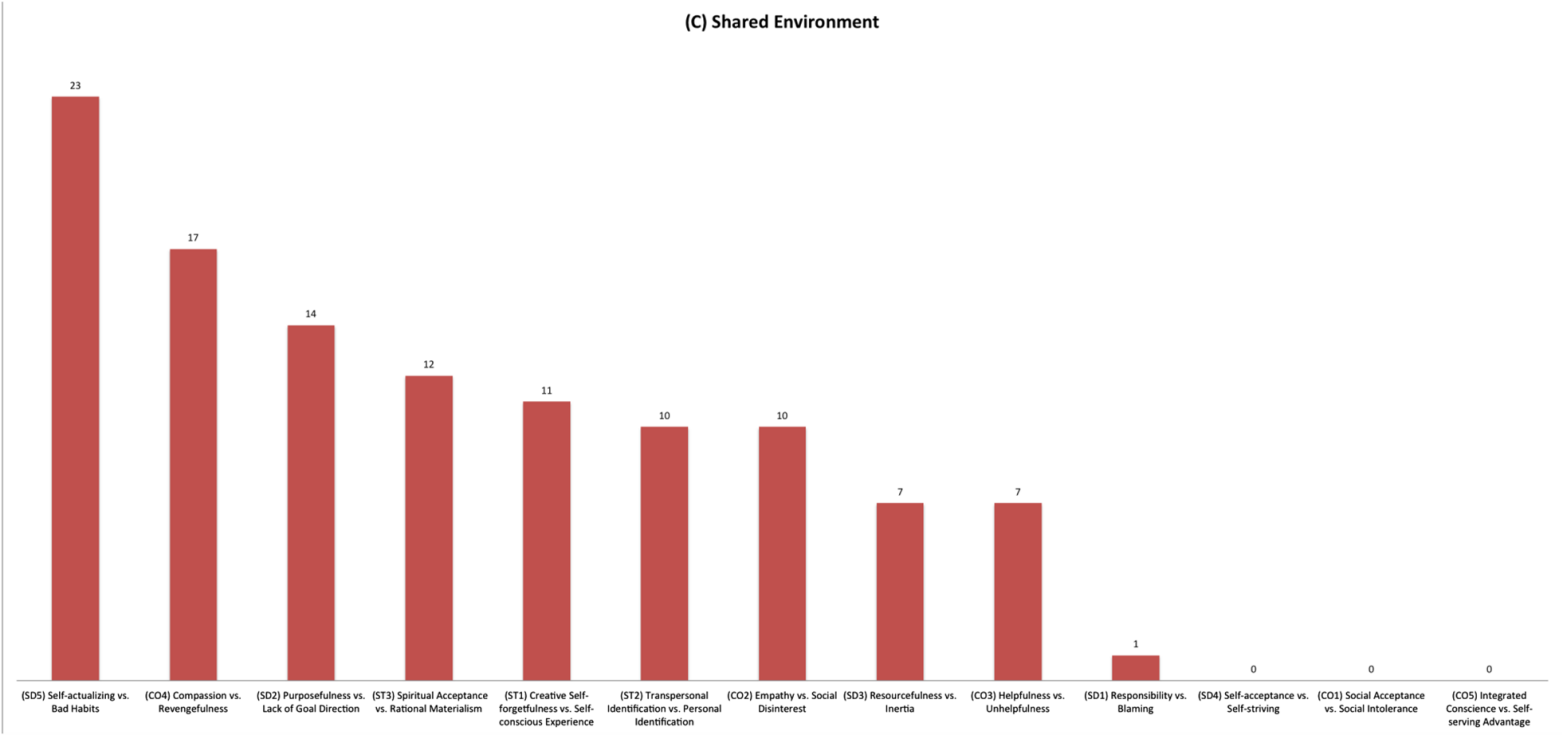
The effect sizes in the present study of shared environment (*C*) across the character lower order sub-scales of self-directedness, cooperativeness, and self-transcendence

The genetic structure at the level of the character lower order sub-scales in adolescents shows that the proportion of the shared environmental component varies among sub-scales of self-directedness and cooperativeness, while it is relatively stable across the trait of self-transcendence (see Figs. 3a, b, 4). We also note that SD4 (self-acceptance vs. self-striving), CO1 (social acceptance vs. social intolerance), and CO5 (integrated conscience vs. self-serving advantage) have no evidence of shared environmental effects. This is also what is observed among adult populations. On the other hand, in adolescents a shared environmental effect is clearly present in all of the lower order sub-scales of self-transcendence and some of the other lower order sub-scales of cooperativeness and self-directedness (see Fig. 5). In particular the common environment influence is substantial for adolescents to develop purposefulness (i.e., SD2), self-actualization (i.e., SD5), and compassion (i.e., CO4). Therefore, it is important to consider how these traits might be related to the processes of socio-cultural learning. We know that character develops in directions that correspond to socially sanctioned norms [28, 29], but we know little about the details of the psychobiological mechanisms by which such socio-cultural learning occurs. However, we also know that individual differences in character traits, measured by the temperament and character inventory, are correlated with variability in the structure and function of particular networks in the human cerebral cortex [30–32]. The processes of purposefulness and self-actualization requires regulating and cultivating particular lifestyle habits consistent with personally chosen goals and values, which requires personal discipline but also may be strongly reinforced or extinguished by cultural effects. Similarly, the development of compassion, forgiving others and not holding grudges, and the development of a purpose have strong cultural connections [33, 34].

A smaller effect size for common environmental influence and social learning is also seen in the sub-scales of self-transcendence (ST1–ST3). This might at first glance seem paradoxical since self-transcendence is often associated with the religious cultural environment [1]. However, while there is clearly an overlap with religious experience and religiosity, self-transcendence is measuring a phenomenon quite distinct from notions of religion, an observation supported by the neurophysiological data [35]. That being said, Magen’s [36] research suggests that adolescents address simple forms of self-transcendence—usually not referring to a macrocosmic unity. Perhaps because adolescents' pursuit of positive emotions tend to be egocentric and directed by their own desires, which in turn is contradictory to the willingness to become dedicated to the well-being of others or pro-social causes that transcend the self [37]. Nonetheless, Magen [36] points out that some adolescents can express transcendent feelings (e.g., mystical identification with a crowd on a strike in the streets) and that even adolescents’ homelier joys uncover “those universals that lead from and go beyond personal experience” (pp. 167). Finally, a slightly smaller size effect size is seen in CO2 (empathy vs. social disinterest), SD3 (resourcefulness vs. inertia), and CO3 (helpfulness vs. unhelpfulness), while SD1 (responsibility vs. blaming) has a very small effect size.

The presence of a shared environmental effect in all character traits in this adolescent sample is suggestive of the greater significance of socio-cultural learning at this critical developmental stage in the human life cycle. Something unique may be taking place not just biologically and psychologically, but also at a socio-cultural level during this phase. The power of cultural reinforcement and the impact of shared narratives may be at its greatest during the adolescent phase of development, compared to adults [38–41]. It may be that in children there is a greater shared environmental effect that is tailing off in adolescence or that the peak period of a shared environmental effect is occurring in adolescence and that the effect sizes might therefore vary for each lower order sub-scale. For instance, Erikson’s stage of identity vs. role confusion [42], which occurs during adolescence, underscores the interaction between the internal drives of identity and socio-cultural awareness of place and identity in the community or environment. To some extent every adolescent must reconcile the identity which she ascertains from the family culture and wider social culture that she happens to be born into with her identity; which is a result from her growing awareness of her individual differences, whether they be relatively common (e.g., being sporty, being tall, being intellectual) or more profound (e.g., being physically different, sexual orientation or, indeed, being a twin).

We are, indeed, beginning to understand how the current models of genetic effects and genetic architecture might be inadequate because they have neglected the cultural inheritance [43, 44] and the complex adaptive processes that are crucial in personality development (e.g., [45]). Personality maturity is itself a complex dynamic system [3, 46]. It is therefore likely that the power of the shared environmental effects across the lifespan is underestimated when complex dynamical patterns of development are neglected.

The evidence we provide for the presence of this unique shared environmental effect in adolescence implies that socio-cultural effects may have implications for understanding the relative importance of interventions and treatment strategies aimed at promoting overall maturation of character. The development of a mature character has been found to correlate with health, happiness, and well-being in the adult human. (e.g., [25]). One of the ways this maturity influences well-being is by the increased ability to temper the emotions. The adolescent is exercising her character’s influence over her temperament in new and important ways, developing her relationship with herself, her fellow humans, and the complex and awe-inspiring universe in which she finds herself. In addition to her self-narrative, the narratives that she is exposed to through the cultural milieu in which she moves will significantly influence this maturation [36, 37, 39–41, 47].

Narratives of unity and connectedness foster the sense of her place in the universe [7]. A culture that fosters tolerance, empathy, and compassion fosters love and cooperativeness. Narratives of responsibility and purpose in life might strengthen her self-exploration in hope and increase her self-directedness. We might consider that in the development of brain connectedness and personality structure, we know the infant draws greatly on the material plasticity of her brain; the adolescent draws in addition the flexibility of her response to and learning from the socio-cultural environment. In later life, as adults mature in character, they may become more self-aware thus increasing the importance of their individual experiences and their expressions of individual virtue in action [3].

### Limitations

It is possible that our findings regarding the genetic structure of Cloninger’s model of personality differ from those of earlier research because of differences in measurement. Most research has been done using the longer version of the Temperament and Character Inventory, but similar results to those obtained with the long version have been found using shorter versions (e.g., Gillespie and colleagues used a 35-item version for measuring the character dimensions). Nonetheless, the short version character scores that were extracted from the clinical sample are highly correlated to their respective long version character scores (see [14]), thus, suggesting that it will produce comparable results when the genetic structure of the model is investigated. In addition, of the 13 character sub-scales only one (SD5) has a 95 % CI for common environmental effects (C) that does not include zero. The estimates of common environmental effects (C) are quite small: 3 are zero, 5 more are .10 or less, and 3 of the remaining 5 are under .15. Such a relatively small shared environmental contributions would seem to provide very little guidance for intervention, except, perhaps, to suggest that the environmental variables currently differing among families in Sweden don’t have much effect on the character traits measured by the sub-scales, so something quite different should be tried if one aspires to change them much. Indeed, well-being interventions recently developed (e.g., well-being coaching; http://www.anthropedia.org/learn-more/) require the development of self-awareness and personality of the whole human being (i.e., body, mind, and psyche or soul^2^).

## Conclusion and final remarks

In thinking about the influence of socio-cultural learning we must consider character development at the lower order sub-scale level: each of the lower sub-scales demonstrates the possibility for an outlook of unity [e.g., being able to show integrity (SD5), to be forgiving (CO4), and creative (ST3)] and an outlook of separation (e.g., undisciplined, revengeful, and judgmental) [52]. The cultural learning environment of the adolescent can support this process of discriminating the two. In the development of self-actualization (SD5) and compassion (CO3) for example, we can see that an outlook of unity might be reinforced by socio-cultural learning experiences. An outlook of unity reinforces the awareness of how our actions have consequences not only for ourself, but also for others and the universe as a whole. With such insight, people become motivated to exercise discipline in changing their daily habits in order to live in accord with their most deeply held values and understanding of their place in the world [3, 7].

On the other hand a narrative of separation will reinforce the almost magical notion that we exist in separation to any consequences, or that consequences themselves do not exist. The balance between our outlooks of separation and unity, therefore, has important and far-reaching implications for happiness, well-being, and mental health. Self-defeating behaviors, often witnessed in adolescence, might continue into adulthood despite evidence of the negative consequences, because the connectedness is not directly understood. New approaches to counteract bullying in schools implicitly acknowledge the importance of this learning. In an outlook of separation, the consequences of the behaviors are rarely appreciated and hardly seem relevant. Approaches based on restorative justice [47], for instance, aim to create a socio-cultural experience for the adolescent allowing them to connect consequences and people affected by her behaviors, thus providing opportunities of learning that integrate values with behavior (see [53]).

*“Harry, I owe you an explanation,ʹ said Dumbledore. `An explanation of an old manʹs mistakes. For I see now that what I have done, and not done, with regard to you, bears all the hallmarks of the failings of age. Youth cannot know how age thinks and feels. But old men are guilty if they forget what it was to be young … and I seem to have forgotten, lately …ʹ”*

Harry Potter and the Order of the Phoenix by J. K. Rowling.

## Additional file

10.1186/s12991-016-0094-2 Correlations between the five lower order sub-scales that compose the self-directedness (SD) scale of the temperament and character inventory (*N* = 2714). **Table S2.** Correlations between the five lower order sub-scales that compose the Cooperativeness (CO) scale of the temperament and character inventory (*N* = 2714). **Table S3.** Correlations between the three lower order sub-scales that compose the self-transcendence (ST) scale of the temperament and character inventory (*N* = 2714).

## Abbreviations

A: additive genetics factors
C: common environmental factors
E: non-shared environmental factors
CATSS: The Child and Adolescent Twin Study in Sweden
MZ: monozygotic
DZ: dizygotic
ICC: intraclass correlation
SD1: responsibility vs. blaming
SD2: purposefulness vs. lack of goal direction
SD3: resourcefulness vs. inertia
SD4: self-acceptance vs. self-striving
SD5: self-actualizing vs. bad habits
CO1: social acceptance vs. social intolerance
CO2: empathy vs. social disinterest
CO3: helpfulness vs. unhelpfulness
CO4: compassion vs. revengefulness
CO5: integrated conscience vs. self-serving advantage
ST1: creative self-forgetfulness vs. self-conscious experience
ST2: transpersonal identification vs. personal identification
ST3: spiritual acceptance vs. rational materialism

## Footnotes

1 “The exact algorithm to select candidates for the questionnaire study was a DSM A-TAC score for attention deficit/hyperactivity disorder ≥8, autism spectrum disorders ≥4.5, conduct ≥1, opposition ≥2, compulsions ≥1, Tics ≥1, eating problems ≥1 and an endorsement of dysfunction and/or suffering related to the symptoms (a problem score of ≥1), or had a parentally reported clinical diagnosis of one or more of these conditions, in total corresponding to 7 % of the children in 13 % of the twin pairs, and a random sample of control twin pairs (1 in 20 interviews). Since November 2008, with access to new validation information, the questionnaires have also been sent to pairs in which one or both twins scored ≥8 in attention deficit/hyperactivity disorder, ≥4.5 in autism spectrum disorders, ≥1.5 in eating problems, ≥3 in oppositional/conduct ≥2 in Tics, ≥1 in compulsions, ≥1 in motor control, or ≥3 in Learning using the DSM score regardless of whether they indicated dysfunction or suffering related to the problems or characteristics” [20].

2 The Greek word *psyche* found in *psych*ology and *psych*iatry stands for "life, soul, or spirit,", which is distinct from *soma*, which refers to the "body" [3]; see also [48–51].
