# Supplementary material for: The genetic and environmental structure of the character sub-scales of the temperament and character inventory in adolescence
Source: Ann Gen Psychiatry. 2016 Mar 12;15:10. doi: 10.1186/s12991-016-0094-2 (PMC4788834; doi:10.1186/s12991-016-0094-2)
Supplement: Supplementary file 1 — 10.1186/s12991-016-0094-2 Correlations between the five lower order sub-scales that compose the self-directedness (SD) scale of the temperament and character inventory (N = 2714). Table S2. Correlations between the five lower order sub-scales that compose the Cooperativeness (CO) scale of the temperament and character inventory (N = 2714). Table S3. Correlations between the three lower order sub-scales that compose the self-transcendence (ST) scale of the temperament and character inventory (N = 2714). [file 12991_2016_94_MOESM1_ESM.docx]

**SUPLEMMENTARY MATERIAL**

**The Genetic and Environmental Structure of the Character Sub-Scales of the Temperament and Character Inventory in Adolescence**

Nigel Lester^1^, E-mail: [nchristopherlester@gmail.com](mailto:nchristopherlester@gmail.com)

Danilo Garcia^1, 2, 3, 4, 5, 6*^, E-mail: [danilo.garcia@icloud.com](mailto:danilo.garcia@icloud.com)

Sebastian Lundström^6, 7, 8^, E-mail: [sebastian.lundstrom@neuro.gu.se](mailto:sebastian.lundstrom@neuro.gu.se)

Sven Brändström^6^, E-mail: [sven.brandstrom@umea.se](mailto:sven.brandstrom@umea.se)

Maria Råstam^9^, E-mail: [maria.rastam@med.lu.se](mailto:maria.rastam@med.lu.se)

Nóra Kerekes^6^, E-mail: [nora.kerekes@neuro.gu.se](mailto:nora.kerekes@neuro.gu.se)

Thomas Nilsson^6, 7^, E-mail: [thomas.nilsson@neuro.gu.se](mailto:thomas.nilsson@neuro.gu.se)

C. Robert Cloninger^1^, E-mail: [crcloninger44@gmail.com](mailto:crcloninger44@gmail.com)

Henrik Anckarsäter^6^, E-mail: [henrik.nckarsater@neuro.gu.se](mailto:henrik.nckarsater@neuro.gu.se)

^1^Department of Psychiatry, Center for Well-Being, Washington University School of Medicine in St. Louis, Missouri, USA

^2^Blekinge Center of Competence, Blekinge County Council, Karlskrona, Sweden

^3^Department of Psychology, University of Gothenburg, Gothenburg, Sweden

^4^Department of Psychology, Lund University, Lund, Sweden

^5^Network for Empowerment and Well-Being, Sweden

^6^Institute for Neuroscience and Physiology, Centre for Ethics, Law and Mental Health (CELAM), University of Gothenburg, Gothenburg, Sweden

^7^Swedish Prison and Probation Service, R&F unit, Sweden

^8^Gillberg Neuropsychiatry Centre, Institution of Neuroscience and Physiology, University of Gothenburg.

^9^Department of Clinical Sciences, Lund, Lund University, Sweden

* Correspondence concerning this article should be addressed to D. Garcia, Network for Empowerment and Well-Being, Axel W. Anderssons Väg 8 A, SE 371 62 Lyckeby, Sweden. E-mail: [danilo.garcia@icloud.com](mailto:danilo.garcia@icloud.com); [danilo.garcia@neuro.gu.se](mailto:danilo.garcia@neuro.gu.se)

**Table S1.** Correlations between the five lower order sub-scales that compose the Self-directedness (SD) scale of the Temperament and Character Inventory (*N* = 2714).

|  | SD | SD1 | SD2 | SD3 | SD4 | SD5 |
| --- | --- | --- | --- | --- | --- | --- |
| (SD) Self-directedness | - |  |  |  |  |  |
| (SD1) Responsibility vs. Blaming | .89*** | - |  |  |  |  |
| (SD2) Purposefulness vs. Lack of Goal Direction | .85*** | .73^***^ | - |  |  |  |
| (SD3) Resourcefulness vs. Inertia | .84*** | .69^***^ | .63^***^ | - |  |  |
| (SD4) Self-acceptance vs. Self-striving | .79*** | .61^***^ | .55^***^ | .55^***^ | - |  |
| (SD5) Self-actualizing vs. Bad Habits | .87*** | .74^***^ | .70^***^ | .70^***^ | .58^***^ | - |
| *Cronbach’s α* | .80 | .83 | .60 | .67 | .79 | .69 |

Note: *** *p* < .001.

**Table S2.** Correlations between the five lower order sub-scales that compose the Cooperativeness (CO) scale of the Temperament and Character Inventory (*N* = 2714).

|  | CO | | CO1 | | CO2 | | CO3 | | CO4 | | CO5 | |  |
| --- | --- | --- | --- | --- | --- | --- | --- | --- | --- | --- | --- | --- | --- |
| (CO) Cooperativeness | - | |  | |  | |  | |  | |  | |  |
| (CO1) Social Acceptance vs. Social Intolerance | .80*** | | - | |  | |  | |  | |  | |  |
| (CO2) Empathy vs. Social Disinterest | .61*** | | .32^***^ | | - | |  | |  | |  | |  |
| (CO3) Helpfulness vs. Unhelpfulness | .77*** | | .56^***^ | | .34^***^ | | - | |  | |  | |  |
| (CO4) Compassion vs. Revengefulness | .81*** | | .59^***^ | | .34^***^ | | .50^***^ | | - | |  | |  |
| (CO5) Integrated Conscience vs. Self-serving Advantage | .77*** | | .54^***^ | | .31^***^ | | .53^***^ | | .54^***^ | | - | |  |
| *Cronbach’s α* | | .72 | | .65 | | .42 | | .41 | | .68 | | .39 | |

Note: *** *p* < .001.

**Table S3.** Correlations between the three lower order sub-scales that compose the Self-transcendence (ST) scale of the Temperament and Character Inventory (*N* = 2714).

|  | ST | ST1 | ST2 | ST3 |
| --- | --- | --- | --- | --- |
| (ST) Self-transcendence | - |  |  |  |
| (ST1) Creative Self-forgetfulness vs. Self-conscious Experience | .85*** | - |  |  |
| (ST2) Transpersonal Identification vs. Personal Identification | .77*** | .48^***^ | - |  |
| (ST3) Spiritual Acceptance vs. Rational Materialism | .83*** | .54^***^ | .47^***^ | - |
| *Cronbach’s α* | .78 | .59 | .47 | .62 |

Note: *** *p* < .001.
